# Intensive Therapeutic Plasma Exchange for Severe Yellow Fever: What Is the Evidence? Comment on Ho et al. Intensive Therapeutic Plasma Exchange—New Approach to Treat and Rescue Patients with Severe Form of Yellow Fever. *Trop. Med. Infect. Dis.* 2025, *10*, 39

**DOI:** 10.3390/tropicalmed10120342

**Published:** 2025-12-08

**Authors:** Till F. Omansen, Michael Ramharter

**Affiliations:** 1 Center for Tropical Medicine, Bernhard Nocht Institute for Tropical Medicine & I Dept. of Medicine University Medical Center Hamburg-Eppendorf, D-20359 Hamburg, Germany; michael.ramharter@ctm.bnitm.de; 2 Department of Virology, Bernhard Nocht Institute for Tropical Medicine, D-20359 Hamburg, Germany; 3 German Center of Infection Research, Partner Site Hamburg-Lübeck-Borstel-Riems, Germany

Recent outbreaks of yellow fever in Brazil, with hundreds of cases despite available vaccination, have drawn attention to the pressing need for effective therapeutic interventions, with a special focus on the critically ill. The most common manifestation of severe yellow fever is rapidly progressive acute liver failure, requiring specialized intensive care to improve survival rates in the absence of effective antiviral or host-directed therapeutics.

In this context, the study by Ho et al. on the use of therapeutic plasma exchange (TPE) in patients with severe yellow fever provides fascinating insights from a highly challenging outbreak situation, which requires careful consideration [1]. TPE, which involves the removal of plasma and its replacement with donor plasma or other fluids, has been evaluated in various liver failure settings, including sepsis, viral infections, autoimmune diseases, and drug overdoses [2]. TPE has shown promise in alleviating the consequences of liver failure by removing harmful toxic substances, including bilirubin, cytokines, and metabolic waste products. This procedure has been advocated as a strategy to bridge patients to liver transplantation in cases where they experience delays in receiving a transplant [3].

The study by Ho et al. investigated the use of TPE for yellow fever patients in Brazil, contributing to this growing body of evidence for the use of TPE. Their findings suggest that intensive TPE may reduce mortality in severe yellow fever patients, with mortality rates significantly lower in the group receiving intensive TPE (14%) compared to those receiving standard intensive care (85%) or high-volume TPE (82%). While this reduction in mortality seems compelling, it must be interpreted with caution due to the observational nature of the study, lacking a randomization process or parallel control group. This retrospective analysis compared treatment protocols that were implemented sequentially over the course of an outbreak. From a methodological point of view, this comparison using historical controls represents a key limitation of the study, introducing a risk of bias. This may include the possibility that the reported improvements in mortality over time were due to factors unrelated to TPE, such as differences in patient selection, improvements in supportive care over the course of the outbreak, or the accumulation of clinical experience as the outbreak progressed.

These limitations point to a larger issue in clinical research in resource-limited settings: conducting a prospective, randomized trial during an outbreak is highly challenging. On the one hand, withholding a potentially life-saving intervention like TPE from a severely ill patient may not be justifiable from an ethical perspective. On the other hand— and from a methodological point of view—the absence of a well-designed trial makes it difficult to establish a clear cause-and-effect relationship between TPE and improved outcomes. While the results of this observational study are promising, they should be viewed as a first step in our understanding of improved management of yellow fever rather than as definitive evidence. Larger, multicenter, prospective trials with randomized controls are needed to firmly establish the efficacy of TPE in severe yellow fever.

Meanwhile, it is also worth considering other potential therapies for severe liver failure in yellow fever patients. One such alternative is albumin-based dialysis, which has been increasingly used in patients with acute liver failure, particularly in cases where TPE is not available or feasible. Albumin-based dialysis systems, such as ADVOS^®^ (by Advito, Hannover, Germany) or MARS^®^ (by Gambro/Baxter, Lund, Sweden), have demonstrated the ability to remove toxins, restore metabolic balance, and support liver function. These therapies also offer the potential to act as a bridge to liver transplantation, similar to TPE [4,5].

The study by Ho et al. offers valuable insight into a novel approach to managing severe yellow fever. The study represents an important contribution to the field, and the authors should be commended for their efforts in advancing our understanding of this life-threatening disease. Further research, including well-designed prospective trials, is necessary to confirm the benefits of TPE and to explore other potential interventions to reduce mortality in severe yellow fever.
